# Spatial effects analysis of natural forest canopy cover based on spaceborne LiDAR and geostatistics

**DOI:** 10.3389/fpls.2024.1361297

**Published:** 2024-07-04

**Authors:** Jinge Yu, Li Xu, Qingtai Shu, Shaolong Luo, Lei Xi

**Affiliations:** ^1^ College of Forestry, Southwest Forestry University, Kunming, China; ^2^ Institute of Ecological Protection and Restoration, Chinese Academy of Forestry, Beijing, China

**Keywords:** spaceborne LiDAR, ICESat-2/ATLAS, geostatistics, natural forests, canopy cover, spatial autocorrelation, spatial heterogeneity

## Abstract

Because of the high cost of manual surveys, the analysis of spatial change of forest structure at the regional scale faces a difficult challenge. Spaceborne LiDAR can provide global scale sampling and observation. Taking this opportunity, dense natural forest canopy cover (NFCC) observations obtained by combining spaceborne LiDAR data, plot survey, and machine learning algorithm were used as spatial attributes to analyze the spatial effects of NFCC. Specifically, based on ATL08 (Land and Vegetation Height) product generated from Ice, Cloud and land Elevation Satellite-2/Advanced Topographic Laser Altimeter System (ICESat-2/ATLAS) data and 80 measured plots, the NFCC values located at the LiDAR’s footprint locations were predicted by the ML model. Based on the predicted NFCC, the spatial effects of NFCC were analyzed by Moran’s *I* and semi-variogram. The results showed that (1) the Random Forest (RF) model had the strongest predicted performance among the built ML models (R^2^=0.75, RMSE=0.09); (2) the NFCC had a positive spatial correlation (Moran’s *I* = 0.36), that is, the CC of adjacent natural forest footprints had similar trends or values, belonged to the spatial agglomeration distribution; the spatial variation was described by the exponential model (C_0_ = 0.12×10^-2^, C = 0.77×10^-2^, A_0_ = 10200 m); (3) topographic factors had significant effects on NFCC, among which elevation was the largest, slope was the second, and aspect was the least; (4) the NFCC spatial distribution obtained by SGCS was in great agreement with the footprint NFCC (R^2^ = 0.59). The predictions generated from the RF model constructed using ATL08 data offer a dependable data source for the spatial effects analysis.

## 1 Introduction

Canopy cover (CC), as a significant forest structure parameter, represents the ratio of vertical canopy projection area to forest area (Lauri et al., 2006), and can reflect the growth and development characteristics of trees and the degree of utilization of growth space.

In the process of forest growth, restoration, and secondary succession, the forest is not only restricted by its site conditions, but also affected by the spatial relationship between the overall structure and other patches in the surrounding areas and these regional characteristics, which leads to a certain spatial effect of the forest (Guo and Zhang, 2002). Neglecting the spatial effects may lead to deviation or error in analyzing and estimating the change pattern of the forest parameters (Anselin and Griffith, 1988; Zhang and Shi, 2004; Stojanova et al., 2013). Spatial effects are commonly described by spatial autocorrelation and heterogeneity (Anselin, 1988; Chen, 2013). Spatial autocorrelation analysis is a widely utilized method in spatial analysis (Legendre, 1993; Cressie and Moores, 2022). It can be classified into two types: global and local spatial autocorrelation (Kashlak and Yuan, 2022; Posa and De Iaco, 2022). Global spatial autocorrelation focuses on analyzing the spatial distribution state and pattern of attribute values of spatial objects in the whole region, and commonly used statistics include the Moran index (Moran, 1950), Getis'G statistics (Getis and Ord, 1992), and Geary’s C index (Geary, 1954). Local spatial autocorrelation can capture local spatial elements' clustering and difference characteristics (Zhang et al., 2023). The main indexes for analyzing local spatial autocorrelation include the local Moran index, local indicators of spatial association (LISA), and Getis' G statistic (Anselin, 1995; Dalposso et al., 2013). These indexes have been extensively employed to enhance the comprehension of forest distribution and accuracy estimation of forest information in forestry (Shi and Zhang, 2003; Chas-Amil et al., 2015; Yin et al., 2018).

Spatial heterogeneity was a critical theoretical issue in ecological research in the 1990s (Tilman et al., 1994), as a common attribute of geographical phenomena, which refers to the uneven distribution of various geospatial attributes in a certain geographical area (Fischer and Getis, 2010; Wang et al., 2016). Spatial heterogeneity analysis has been widely applied to spatiotemporal problems in ecology, geology, public health, economy, environment, and other fields (Song et al., 2020). Its goals usually include: exploring the spatial aggregation of regions defined as high or low spatial values (Anselin, 1995); analyzing the potential factors leading to the uneven spatial distribution (Brunsdon et al., 1996; Fotheringham et al., 2003); spatiotemporal prediction and decision-making based on spatial heterogeneity (Wang et al., 2014). Gaining a complete comprehension and utilization of spatial heterogeneity can enhance our understanding of forest vegetation growth and the evolution of forest ecosystems (Hewitt et al., 2007; Gossner et al., 2013; Detto et al., 2015; Getzin et al., 2017).

Currently, spatial attributes obtained through remote sensing has become more accessible, facilitating the spatial effects analysis at the regional level. However, in many RS technologies, optical remote sensing does not provide forest vertical structure information and is susceptible to weather and saturation effects (Chopping et al., 2008; Peduzzi et al., 2012; Wang et al., 2019a). Microwave remote sensing can acquire forest information regardless of weather conditions, but it is vulnerable to terrain and saturation issues (Vatandaşlar and Abdikan, 2022). Encouragingly, Spaceborne LiDAR technology can penetrate the canopy to obtain three-dimensional information of vegetations, and has incomparable advantages in large-scale forest structure observation research due to its large-area, multi-scale, and multi-space-time monitoring capabilities and low cost of data acquisition for user (Disney, 2019; Pitkänen et al., 2019; Wang et al., 2019b).

NASA launched the Ice, Cloud, and land Elevation Satellite-2 (ICESat-2) in 2018 as a successor to the Ice, Cloud, and land Elevation Satellite (ICESat). Equipped with the Advanced Topographic Laser Altimeter System (ATLAS), ICESat-2 utilizes multi-beam, micropulse, and photon-counting lidar technology (Magruder and Brunt, 2018). It uses a single photon detector that is more sensitive, has a higher pulse repetition rate, and can obtain observations with more minor spots and higher density. The data have been successfully used to characterize canopy cover. For example, Narine et al (Narine et al., 2022). have tried to estimate the CC by combining the ICESat-2 data, passive optical image, the National Land Cover Database (NLCD) cover product estimates. In comparison to CC derived from airborne LiDAR, The RF models demonstrated R^2^ values ranging from 0.50 to 0.61, with corresponding RMSEs between 0.16 and 0.20. Although these studies demonstrated the power of ICESat-2 to estimate CC, the spatial effects of CC were not further explored.

Remote sensing modeling plays a crucial role in estimating CC and explaining the correlation between remote sensing variables and CC (Chopping et al., 2012; Khokthong et al., 2019; Eskandari et al., 2020; Huang et al., 2021; Miranda et al., 2021). Machine learning approaches provide more general categories, such as decision trees (CART, RF), k-NN, Neural Networks, SVM etc. for CC estimation (Joshi et al., 2006; Ahmed et al., 2015b, Ahmed et al., 2015a; Zhao et al., 2018; Nasiri et al., 2022b). However, it is difficult for an ML algorithm to perform optimally in every study object or area. For example, Zhang et al (Zhang et al., 2022). compared the performance of 6 ML models (k-NN, Gradient Boosting Regression Tree (GBDT), XGBoost, CatBoost, SVR, and RF) in mapping forest heights using multi-source RS data, the optimal performance model is CatBoost. Nasiri et al (Nasiri et al., 2022a). compared the performance of 4 ML algorithms [RF, SVM, ENet and extreme gradient Boost (XGBoost)] in estimating the CC of mixed temperate forests in northern Iran, and the results showed that RF is the best prediction model among the ML algorithms. Pourshamsi et al (Pourshamsi et al., 2021), based on polarimetric SAR and airborne LiDAR data, used 4 ML models (RF, Rotation Forest (RoF), Canonical Correlation Forest (CCF) and SVM) to estimate the forest canopy height of Lope National Park in central Gabon, the SVM performed slightly better. Shu et al (Shu et al., 2022). found that the RF model had the highest R^2^ value (R^2^ = 0.85) among the models of AGB estimation based on the optimal samples. In the above research, researchers find a relatively optimal model by comparing the machine learning. However, the CC estimation still requires the addition of newer models on the basis of general models for comparison to obtain the optimal model. In Addition, based on the advantages of spaceborne LiDAR mentioned earlier, a regional scale spatial effects analysis framework that avoids the shortcomings of optics and SAR is needed for scientific management of forests.

This study provided an alternative for the large-scale spatial effects analysis and a reference for the scientific management of natural forests. Based on ICESat-2/ATLAS data and the four machine learning algorithms, combined with the measured NFCC data, the machine learning models of NFCC were established and evaluated, and then the NFCC values within footprints were predicted by the model with best-predicted performance. Finally, based on the predicted NFCC values, the spatial effects were analyzed. Therefore, this study aimed to: (1) evaluate the performance of different machine learning algorithms in predicting footprint NFCC; (2) describe the spatial heterogeneity and autocorrelation of NFCC at the regional scale; (3) evaluate the influences of elevation, slope, and aspect on the spatial heterogeneity of NFCC; (4) explore suitable interpolation method based on the NFCC values within the footprints.

## 2 Materials and methods

### 2.1 Study area

**Figure 1:**
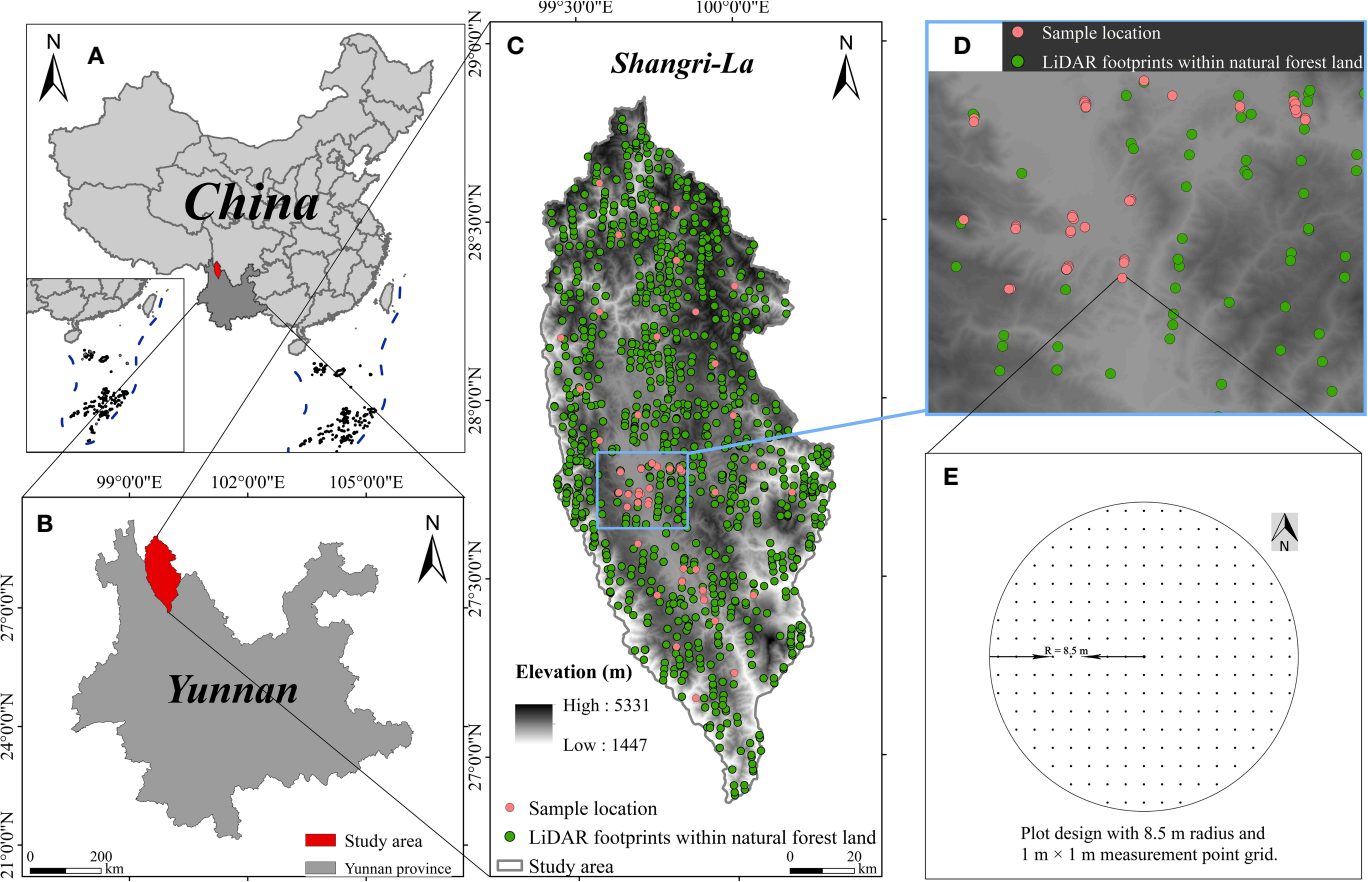
Overview of the study area: **(A)** location of Shangri-La in China, **(B)** location of Shangri-La in Yunnan Province, **(C)** location of LiDAR footprint and ground-truth sample, **(D)** the magnified view of an area, and **(E)** diagram of sample plot design.

The research area is Shangri-La City (**Figure 1**), Diqing Tibetan Autonomous Prefecture, Yunnan Province, China (Latitude: 26°52′~28°52′N, Longitude: 99°20′~100°19′E). The area has significant changes in altitude and is a key forest area, ecological protection area, and tourist area. The dominant tree species include Spruce (*Picea asperata*), Fir (*Abies fabri*), Oak (*Quercus semecarpifolia*), Pinus (*Pinus yunnanensis*), etc (Xu et al., 2021).

### 2.2 Methodological framework

**Figure 2:**
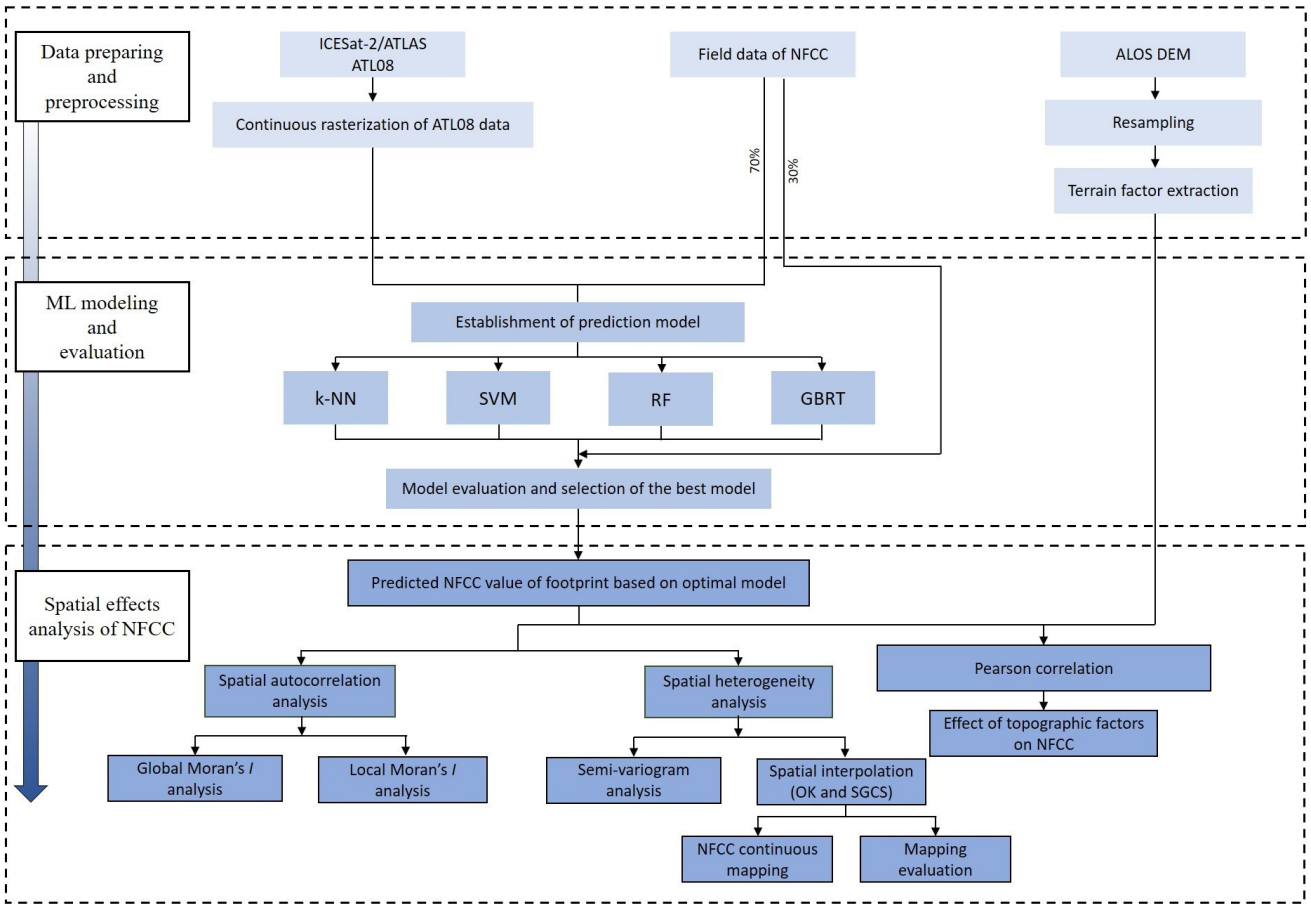
Flowchart for NFCC spatial effects analysis combining the ICESat-2/ATL08 data, field data, and ML modeling.

In this study, four ML algorithms were used to build the estimation model of NFCC based on light spot footprints, and then the NFCC of all light spot footprints was predicted and used as a spatial attribute of spatial heterogeneity analysis. Our framework approach comprises three main components (**Figure 2**): (1) the process of preparing and preprocessing data, including data preprocessing for ATL08, resampling of terrain factors and extraction of slope and aspect; (2) NFCC model construction and evaluation based on four ML models (k-NN, SVM, RF, GBRT), and (3) NFCC spatial effects based on semi-variogram function and terrain impact analysis based on Pearson correlation.

### 2.3 Data source and preprocessing

#### 2.3.1 Field data

Circular plots were established in the study area in November 2021 (**Figure 1C**). Given that ATLAS generates footprints with an approximate diameter of 17 m on the ground (Neumann et al., 2019), the plot was set as a circle with a radius of 8.5m (**Figure 1E**). CC was measured by systematically setting *N* observation points in the sample site to determine whether each observation point was covered by vertical canopy projection. Sight tubes with leveling bubbles were used to reduce measurement bias for non-vertical aiming. The layout of observation points in the circular plot is shown in **Figure 1E**. The formula [Equation (1)] for calculating the CC in the sample plot is as follows (Jennings et al., 1999), and the measurement results of all sample plots are shown in **Table 1**.

**Table 1:** Descriptive statistics of the measured NFCC of the plot.

Item	Sample number	Max.	Min.	Mean	Standard Deviation	Acquisition time
NFCC	80	0.85	0.20	0.52	0.16	Nov. 2021


(1) $$Cc=\frac{m}{M}$$

where: Cc is the value of the CC; M is the number of sample points; *m* is the number of sample points covered by canopy.

#### 2.3.2 ICESat-2/ATL08 product and preprocessing

ATL03, as the basic data for generating other products, provides geospatial information such as the time, ellipsoid height, longitude, and latitude of each photon event (Huang et al., 2020). The ATL08 (Land and Vegetation Height) product, as the primary data source of this study, is officially released by NASA on the basis of ATL03 (Global Geolocated Photons) product after pretreatment, which provides information on terrain and forest canopy height in the track direction.

This study used ATL08 data products within one year after June 1, 2020. There are 11,060 footprints in the natural forest land of study area. Ultimately, 1106 footprints (**Figure 1C**) obtained through systematic sampling (sampling interval = 10) were used as selected footprints for follow-up research.

**Figure 3:**
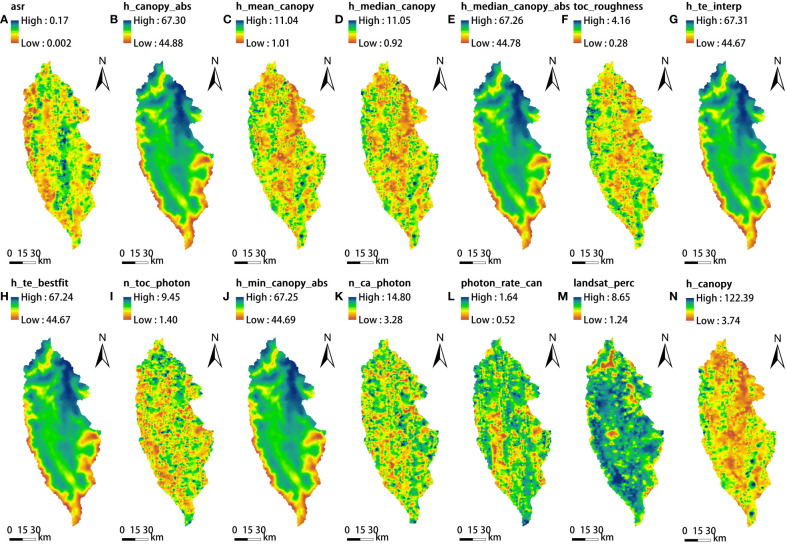
ATL08 continuous rasterization: **(A)** asr, **(B)** h_canopy_abs, **(C)** h_mean_canopy, **(D)** h_median_canopy, **(E)** h_median_canopy_abs, **(F)** toc_roughness, **(G)** h_te_interp, **(H)** h_te_bestfit, **(I)** n_toc_photon, **(J)** h_min_canopy_abs, **(K)** n_ca_photon, **(L)** photon_rate_can, **(M)** landsat_perc, and **(N)** h_canopy.

The ATL08 data presents a spatially discrete footprint resulting in discontinuity of its data. In order to obtain the continuous coverage of ATL08 data on the sample site, based on the parameters of the ATL08 products, normality test was carried out first. Parameters, either initially normal or normalized through data transformation, were subjected to Kriging interpolation and subsequently output as raster layers with a 17 m spatial resolution. Ultimately, the continuous rasterization layers of the normal parameters of ATL08 product are shown in the **Figure 3**.

#### 2.3.3 Topographic data

**Figure 4:**
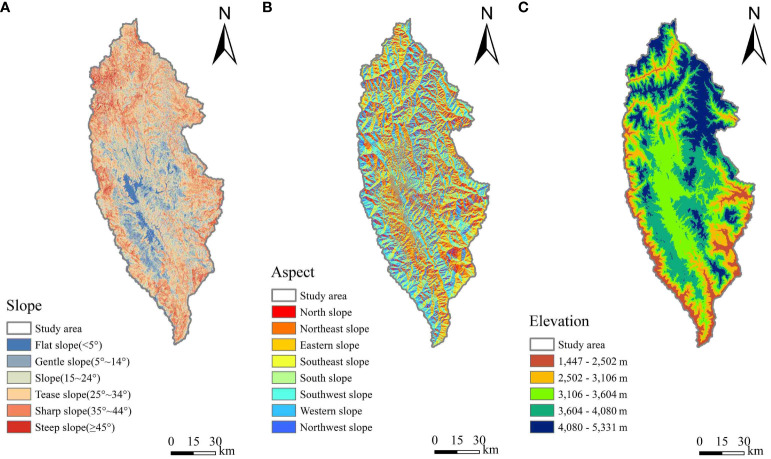
Overview map of topographic factors in the study area: **(A)** slope, **(B)** aspect, and **(C)** elevation.

The DEM data (12.5 m) was obtained by the PALSAR sensor of the Advanced Land Observing Satellite-1 (ALOS) Satellite. With the help of ArcMap 10.8, the DEM data was resampled to a spatial resolution of 17 m to match the ground footprint size, then the aspect and slope were calculated using the 3D analysis toolbox, as shown in **Figure 4**.

#### 2.3.4 National Forest Management Inventory data

The National Forest Management Inventory (FMI) data provides abundant information such as land class, forest species, ownership, forest protection grade, origin, dominant tree species, CC, average stock, etc. The footprints in the natural forest area were extracted using the survey data of the study area in 2016. The natural forests were identified based on their origin, and forests evolution usually take a long time. More importantly, China launched the Natural Forest Conservation Project in 1999 and National Ecological Vulnerable Area Protection Plan in 2008. Therefore, although there is a time lag, the scope of natural forests remains basically unchanged, and still is applicable in this study.

### 2.4 Correlation analysis

In statistics, the Pearson correlation coefficient measures the linear correlation between two variables, with its value ranging from -1 to 1. The closer the coefficient’s absolute value is to 0, the weaker the linear correlation between the two variables. Conversely, an absolute value closer to 1 indicates a stronger linear correlation. The basic principle of Pearson correlation can be seen in the article of Yang et al (Yang et al., 2021). In this study, the correlation analysis was used to screen model independent variables and explain the effect of terrain factors on NFCC.

### 2.5 Machine learning methods

k-nearest neighbor (k-NN), a simple and efficient non-parametric method, effectively circumvents the issue of collinearity among independent variables. This algorithm is applicable to the parameter estimation of remote sensing data characterized by non-normal distributions and unknown density functions, and is extensively utilized in forestry investigations globally (Chirici et al., 2008, 2016). The fundamental concept of this algorithm involves using a point in the feature space as the reference object, capturing the attribute values of the *k* nearest sample points relative to this point, and determining the predicted value of this object by calculating the average of its inverse distance weights.

Support vector machine (SVM) algorithm originates from the VC dimension theory and the structural risk minimization principle (Chirici et al., 2008, 2016). The fundamental principle of SVM involves mapping training data features to a high-dimensional space through a defined kernel function, and identifying an optimal linear regression hyperplane in this space that best fits the eigenvalues.

RF proposed by Breiman (Breiman, 2001), is a method that combines weak classifiers to create strong classifiers. Its fundamental concept lies in the ability of the ensemble to compensate for incorrect predictions made by individual weak classifiers. Originally developed as an extension of classification and regression trees (CART), RF enhances predictive models by generating aggregate predictors (Breiman, 1996).

Gradient Boost Regression Tree (GBRT), as an ensemble learning method, builds a strong learning model by sequentially aggregating a set of weak CART regression tree submodels (Opitz and Maclin, 1999; Friedman, 2001). The key concept of GBRT is that each new regression tree submodel is built in the gradient direction of residuals reduction to reduce residuals from previous models (Liu et al., 2020).

In our research, we randomly divided the plot data into two sets: training dataset (70%) and validation dataset (30%). The training set served to train and develop the models, whereas the validation set, not participating in the model-building process, was used to evaluate model performance. Root mean square error [RMSE; Equation (2)] and coefficient of determination [(R^2^; Equation (3)], as two commonly used evaluation indexes, were used to evaluate the prediction performance of regression models. A higher R^2^ value indicates greater model accuracy, while a lower RMSE value signifies enhanced accuracy of the regression model. The calculation formulas for each indicator are as follows:


(2) $$RMSE=\sqrt{\frac{\sum_{i=1}^{n}\left(y_{i}-{\hat{y}}_{i}\right)}{n-1}}$$

(3) $$R^{2}=1-\frac{\sum_{i=1}^{n}{\left(y_{i}-{\hat{y}}_{i}\right)}^{2}}{\sum_{i=1}^{n}{\left(y_{i}-{\bar{y}}_{1}\right)}^{2}}$$

where n is the number of samples, ${\hat{y}}_{i}$ is the predicted by the ML models, $y_{i}$ is the observed FCC from the ground measurements, $\bar{y}$ is the arithmetic mean of observed values.

### 2.6 Spatial autocorrelation analysis

Moran’s *I* can effectively capture differences and correlations in the spatial distribution of observations, as well as reflecting the overall clustering pattern of objects in the study area (Zhang et al., 2023). The value interval of Moran’s *I* is [-1, 1]. When the value is less than 0, spatial objects have a negative correlation; when the value is equal or close to 0, spatial autocorrelation does not exist. When the value is greater than 0, it indicates that there is spatial autocorrelation, and spatial objects show a clustered distribution (Moran, 1950). Moran’s *I* formula [Equation (4)] is as follows:


(4) $$\begin{array}{c} Moran^{\prime }s\;I=\left(\frac{n}{\sum_{p=1}^{n}\sum_{q=1}^{n}w_{pq}\left(d\right)}\right)\;\frac{\sum_{p=1}^{n}\sum_{q=1}^{n}w_{pq}\left(d\right)\left(x_{p}-\bar{x}\right)\left(x_{q}-\bar{x}\right)}{\sum_{p=1}^{n}{\left(x_{i}-\bar{x}\right)}^{2}} \end{array}$$

where n is the number of observed values; $\bar{X}$ is the average of the variable X ; $x_{p}$ and $x_{q}$ refer to the observation values at plot p and plot q , respectively; $w_{pq}\left(d\right)$ is the spatial weight matrix value between plot p and plot q .

After calculating the Moran’s *I* value, the significance of Moran’s *I* can be tested by a Z-score. A positive Z-score points to a cluster of high values, whereas a negative Z-score suggests clusters of low values. The degree of clustering is greater (or lesser) with a higher Z-score. Conversely, if the Z-score is close to zero, it indicates the absence of significant clustering in the area. Equation (5) was used to calculate the Z-score.


(5) $$\begin{array}{c} Z\left(S\right)=\frac{S-E\left(S\right)}{\sqrt{Var\left(S\right)}},\;E\left(S\right)=\frac{-1}{m-1} \end{array}$$

where Z(S) represents an index that measures the intensity of a spatial agglomeration pattern; E(S) denotes the expected value of the index value I, while Var(S) represents its variance.

Furthermore, local spatial autocorrelation can elucidate the level of spatial correlation existing between a research object and its neighboring units. Equation (6) was used to calculate the local Moran"s *I*.


(6) $$\begin{array}{c} I_{l}=\frac{m^{2}}{\sum_{p}^{m}\sum_{q}^{m}w_{pq}}\frac{\left(x_{p}-\bar{x}\right)\sum_{q}^{m}w_{pq}\left(x_{q}-\bar{x}\right)}{\sum_{p}^{m}{\left(x_{p}-\bar{x}\right)}^{2}} \end{array}$$

where m is the number of plots; $x_{p}$ and $x_{q}$ are the observation values at plot p and plot q , $\bar{x}$ is the average of all NFCC values; $w_{pq\;}\left(d\right)$ is the weight matrix value. The local Moran’s *I* differs from the global Moran’s *I* in terms of value range. Unlike the global Moran’s *I*, the local Moran’s *I* is not limited to the range of (-1, 1). If the $I_{l}$ value is positive, it indicates a positive correlation in the location of the plot and reflects the aggregation of similar values. Conversely, if the $I_{l}$ is negative, it signifies a negative correlation in the location of the plot and reflects the aggregation of different values.

### 2.7 Semi-variogram analysis

Semi-variogram is often used to describe spatial heterogeneity (Wang et al., 2000). In the semi-variogram function parameter, nugget (C_0_) reflects the possible degree of randomness within the regionalized variable, and explains the discontinuous variation of the regional variable at a small scale, which is due to the measurement error and random variation of the sampling scale. Sill (C_0_+C) was used to measure the degree of spatial heterogeneity and reflected the maximum degree of variation of the variable. Range (a) refers to the average maximum distance of spatial autocorrelation between variables (Chiles and Delfiner, 2012). The semi-variogram formula [Equation (7)] (Chiles and Delfiner, 2012) is as follows:


(7) $$r(h)=\frac{1}{2N(h)}\sum_{i-1}^{N(h)}{\left(Z(x_{i})-Z(x_{i}+h)\right)}^{2}$$

where r(h) is the semi-variogram of NFCC; N(h) is the total logarithm of sample points spaced h in one direction; $Z\left(x_{i}\right)$ is the measured NFCC at spatial position $x_{i}$ ; $Z\left(x_{i}+h\right)$ is the NFCC value at h distance from point $x_{i}$ .

The relationship between the semi-variogram value and the distance usually requires a mathematical model to fit. The common mathematical models include spherical model, exponential model, Gaussian model, and linear model. According to the principle that the R^2^ is large and the RSS is tiny, it is found that the exponential model is more suitable for revealing the spatial heterogeneity of the NFCC. The expression of the exponential model [Equation (8)] is as follows:


(8) $$\gamma \left(h\right)=\{\begin{array}{ccc} 0 & , & h=0\\ {\text{C}}_{0}+\text{C}\;\left(1-e^{-\frac{h}{a}}\right) & , & h>0 \end{array}$$

where γ(h) is the semi-variogram of NFCC; a is the range; C is the partial sill value; C_0_ is nugget value; h is distance.

Spatial heterogeneity is not only related to scale, but also to direction (Li and Reynolds, 1995), and the spatial distribution of NFCC is also different depending on the direction. Anisotropic semi-variogram was used to analyze the direct change of spatial heterogeneity of NFCC (Habin et al., 1998). In general, the anisotropy ratio [ K(h) ] between the semi-variogram functions in different directions is used to describe the anisotropic structural characteristics, and the formula [Equation (9)] is as follows:


(9) $$\begin{array}{c} K\left(h\right)=\frac{\lambda \left(h,\;\theta_{1}\right)}{\lambda \left(h,\;\theta_{2}\right)} \end{array}$$

where $\lambda \left(h,\theta_{1}\right)$ and $\lambda \left(h,\;\theta_{2}\right)$ are the semi-variogram functions in the directions $\theta_{1}$ and $\theta_{2}$ , respectively. If K(h) is equal to or close to 1, the spatial heterogeneity is isotropic, otherwise it is anisotropic.

### 2.8 Spatial interpolation method

Ordinary Kriging (OK), its essence is to infer the regionalized spatial distribution of variables by the variable in the spatial regionalization of a finite number of sample points. Based on the information of several measured points in the search field where the point to be estimated is the center of the circle, it uses the semi-variogram function as a tool to calculate the weighted value of the measured points around the point to be estimated, and finally makes the optimal and unbiased estimation of the estimated points (Christakos, 2000). The formula [Equation (10)] is as follows:


(10) $$\begin{array}{c} Z_{e}^{\#}\left(x_{0}\right)=\sum_{i=1}^{m}\lambda_{i}Z\left(x_{i}\right) \end{array}$$

where $Z_{e}^{\#}\left(x_{0}\right)$ represents the predictive NFCC at the point to be predicted; $Z\left(x_{i}\right)$ stands for the observed NFCC at the point to be predicted; $\lambda_{i}$ represents the weight of each known parameter value, and m represents the number of spot footprints.

Sequential Gaussian Conditional Simulation (SGCS) is a spatial stochastic simulation method that constructs a Gaussian function based on known data and treats each value of the regionalized random variable Z(x) as a random realization of the Gaussian function (i.e. the normal distribution function) F(x). It is mainly used to generate spatial explicit estimates of interest variables based on regionalized variable theory and spatial autocorrelation theory measured by the semi-variogram functions. More information and detailed processes about the SGCS can be found in the articles by Luo et al (Luo et al., 2023), and Zhao et al (Zhao et al., 2010).

Zhao et al (Zhao et al., 2010). derived the connection between the OK and the SGCS, and compared the statistical parameters of both computational results and the original data. The results showed that the values from SGCS actually consist of two parts: one is the result of Kriging interpolation, and the other is a random deviation with a mathematical expectation of 0 and a variance equal to the Kriging error variance S (X_m_). The difference lies in that Kriging interpolation solely uses the original known point data as a basis for estimating unknown points, whereas in SGCS, each simulated value for a location not only applies known point data but also previous simulation data. In this section, two interpolation methods, Kriging and SGCS, are applied. The motive is to find a suitable interpolation method for the footprint NFCC obtained by inversion.

In the evaluation of interpolation results, we randomly divided the footprint NFCC predicted by the optimal model into two sets: interpolation dataset (80%) and validation dataset (20%). The interpolation dataset is used for spatial interpolation, and the validation dataset is used to evaluate the interpolation results.

### 2.9 Software environment

With the help of SPSS 27.0, Pearson correlation analysis was used to evaluate the correlation between ATL08 parameters and NFCC, and the ATL08 parameters were selected as independent variables of the model according to the value and significance of the correlation coefficients.

Based on Python 3.7 environment, the four machine learning algorithms (k-NN, SVM, RF, GBRT) in this study were implemented using the scikit-learn package in the Python library.

Global and local Moran’s *I* were calculated in the spatial analysis toolbox of ArcGIS 10.8.

**Figure 5:**
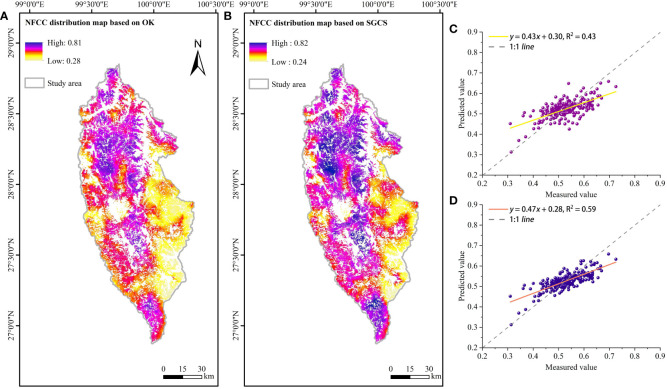
Spatial distribution mapping and evaluation: **(A)** NFCC spatial interpolation based on OK, **(B)** NFCC spatial interpolation based on SGCS, **(C)** NFCC scatter plot based on OK, and **(D)** NFCC scatter plot based on SGCS.

GS^+^ 9.0 (GeoStatistics for the Environmental Sciences, version 9.0), a comprehensive geostatistics program, provides all geostatistics components, from variogram analysis through Kriging and mapping, in a single integrated program that is widely praised for its flexibility and user-friendly interface. The semi-variogram analysis and the corresponding parameters (C_0_, C_0_+C, a) were obtained in this software. To further know the spatial distribution of NFCC within the study area, based on the NFCC of ATLAS footprints and the fitted semi-variogram function, GS^+^ 9.0 and ArcGIS 10.8 were used to achieve the OK interpolation and SGCS for NFCC (**Figures 5A, B**). The number of SGCS simulations was set to 50 times (Luo et al., 2023).

## 3 Results

### 3.1 Selected ATL08-derived features and the ML modeling

**Figure 6:**
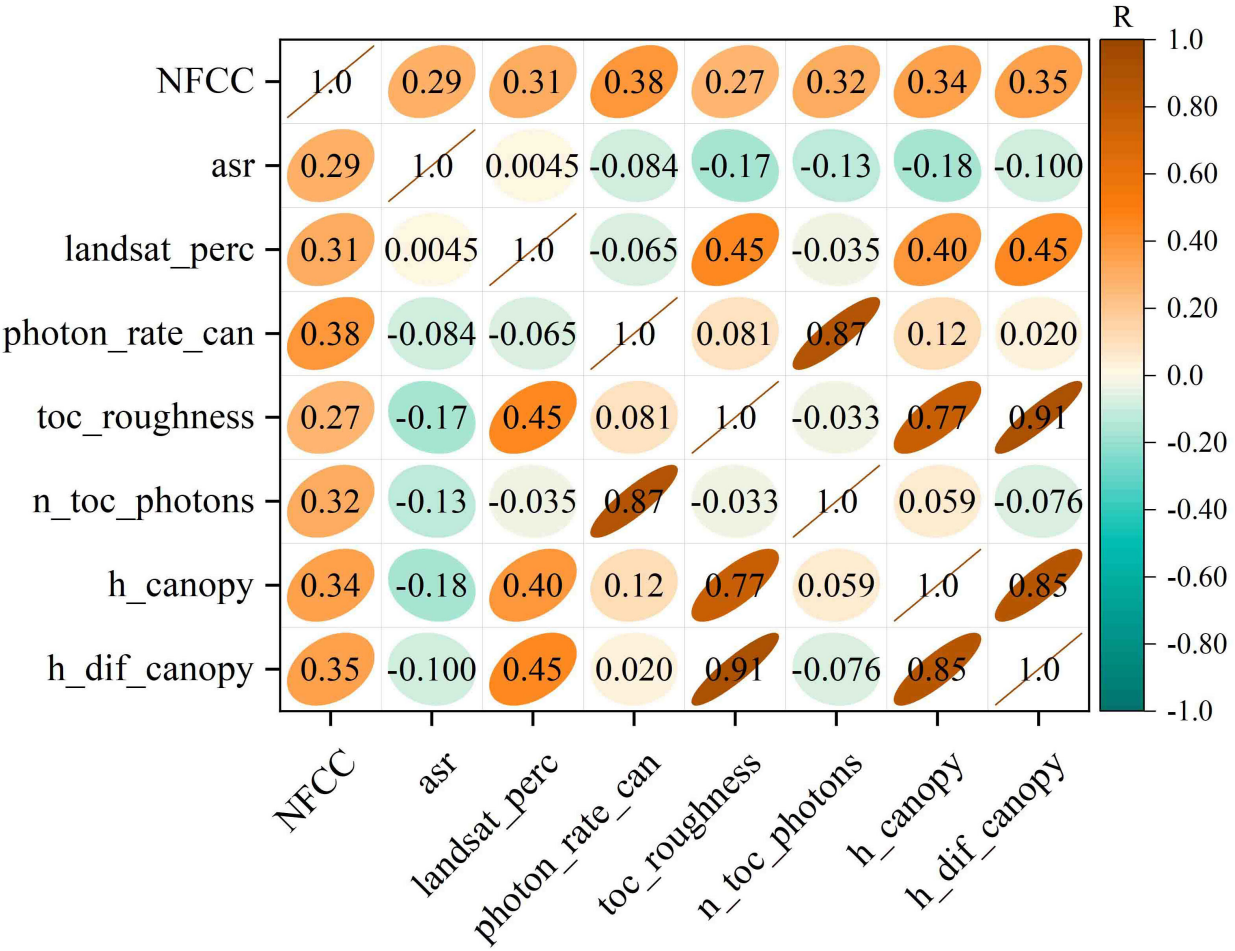
Correlation coefficient matrix between the ATL08 seven parameters and NFCC.

The result of Pearson correlation analysis showed seven parameters from the ATL08 product (asr, landsat_perc, photon_rate_can, toc_roughness, n_toc_photons, h_canopy, h_dif_canopy) were significantly correlated with NFCC at the 0.05 confidence level (**Figure 6**). Then the seven parameters were selected as the independent variables (the description is shown in **Table 2**), and the NFCC measurement serves as the dependent variable for constructing the ML models.

**Table 2:** The correlation coefficient and description of seven ATL08 parameters.

Parameter	Coefficient	Description
asr	0.29	Surface reflectance.
landsat_perc	0.31	Landsat canopy percentage.
photon_rate_can	0.38	Canopy photon ratio.
toc_roughness	0.27	The standard deviation of the relative height of all photons classified as the top of the canopy within the segment.
n_toc_photons	0.32	The number of photons on top of the canopy.
h_canopy	0.34	98% height of all the individual canopy relative heights for the segment above the estimated terrain surface.
h_dif_canopy	0.35	Difference between crown height and median crown height.

**Figure 7:**
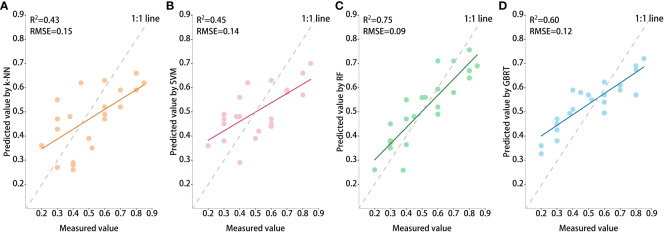
The NFCC models estimation results using the validation dataset: **(A)** k-NN, **(B)** SVM, **(C)** RF, and **(D)** GBRT.

Two statistical metrics (R^2^, RMSE) were applied to evaluate the models constructed, utilizing the reserved 30% of field plot data (**Table 3**). The predicted performance of the ML models was ranked as follows (descending order): RF (R^2^ = 0.75, RMSE = 0.09), GBRT (R^2^ = 0.60, RMSE = 0.12), SVM (R^2^ = 0.45, RMSE = 0.14), k-NN (R^2^ = 0.43, RMSE = 0.15). **Figure 7** showed the comparison of the NFCC predicted values with the measured values in test set.

**Table 3:** Model evaluation parameters.

Regression method	R^2^	RMSE
k-NN	0.43	0.15
SVM	0.45	0.14
RF	0.75	0.09
GBRT	0.60	0.12

### 3.2 Mapping of NFCC and descriptive statistics within footprints

**Figure 8:**
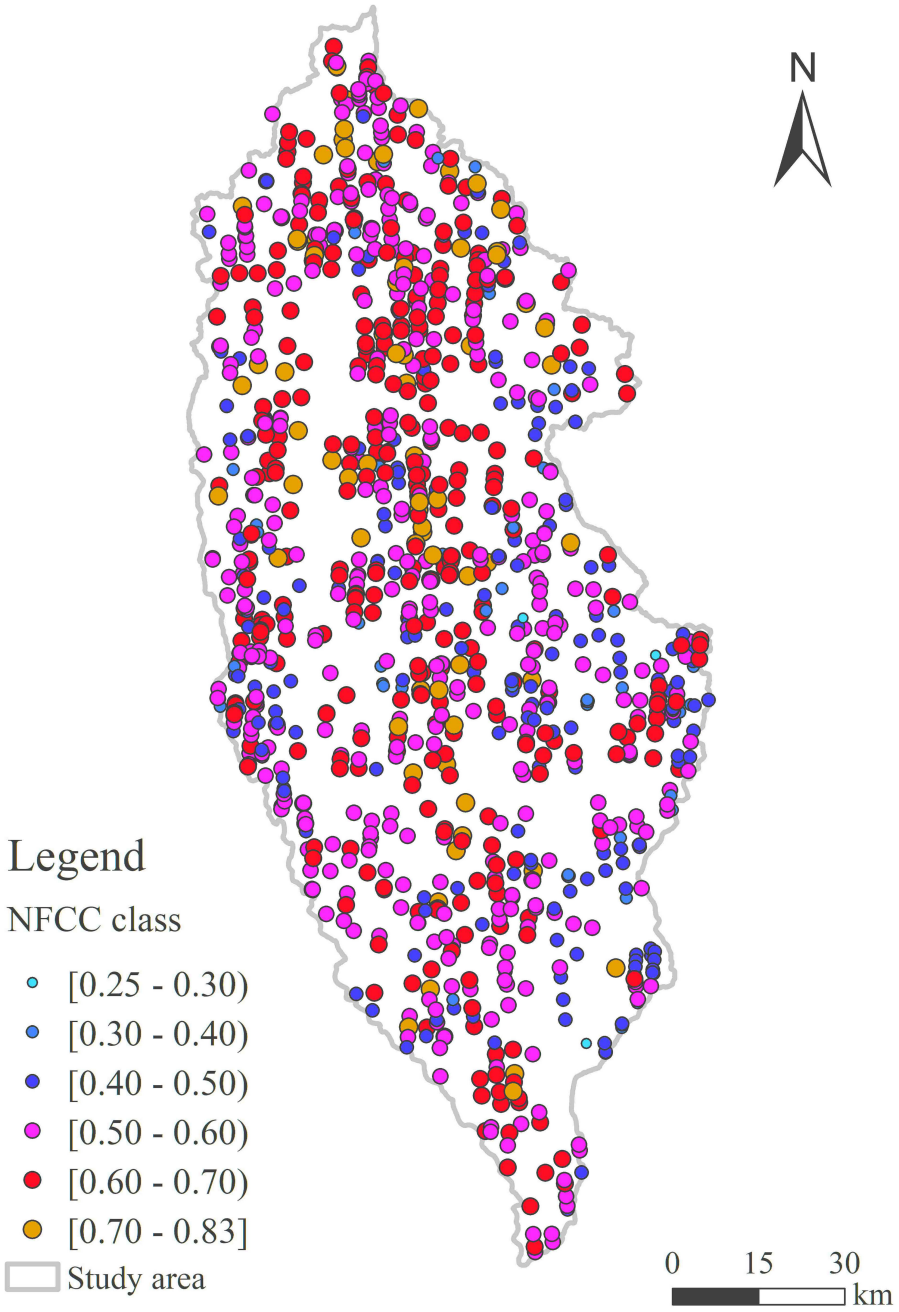
Mapping of footprint NFCC in the study area.

Due to its greater predicted performance, the RF model was used to predict NFCC within the natural forest footprint, and then the footprint NFCC was visualized in **Figure 8**. Most of the natural forest footprint CC was above 0.5. The areas with high-values were mainly distributed in the northwest, middle and south of Shangri-La (**Figure 8**).

**Figure 9:**
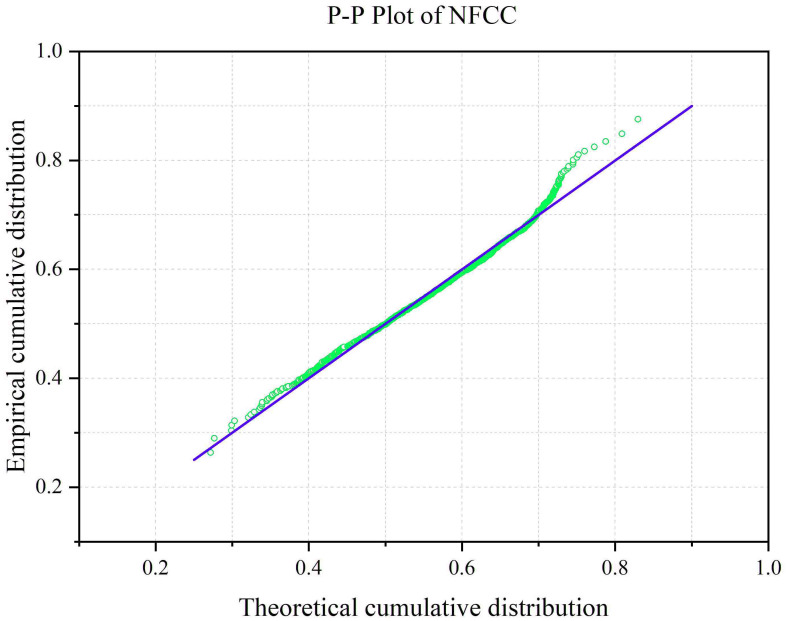
P-P Plot of NFCC within footprints.

**Table 4** showed the footprint’s descriptive statistics of NFCC and topographic factors within the footprint. The P-P Plot of NFCC (**Figure 9**) showed a normal distribution, which meets the requirements of structural analysis of semi-variogram.

**Table 4:** Descriptive statistics of NFCC and topographic factors within the footprint.

Item	Number	Max.	Min.	Mean	Standard deviation
NFCC	1106	0.83	0.25	0.56	0.092
Elevation	1106	4646	1821	3453.88	571.88
Slope	1106	57.34	2.43	26.94	11.06
Aspect	1106	9	1	5.68	2.29

### 3.3 Spatial autocorrelation of NFCC

**Table 5** showed that Z-score = 6.47 and P value< 0.01, indicating that Moran’s *I* passed the test with 99% confidence level. Moran’s *I* of NFCC in the study area is positive (Moran’s *I* = 0.36), indicating that the NFCC has a positive spatial correlation and belongs to spatial agglomeration distribution.

**Table 5:** Moran’s I coefficient of NFCC.

Item	Moran’s *I*	Z score	P value
NFCC	0.36	6.47	P ≈ 0.000 < 0.01

**Figure 10:**
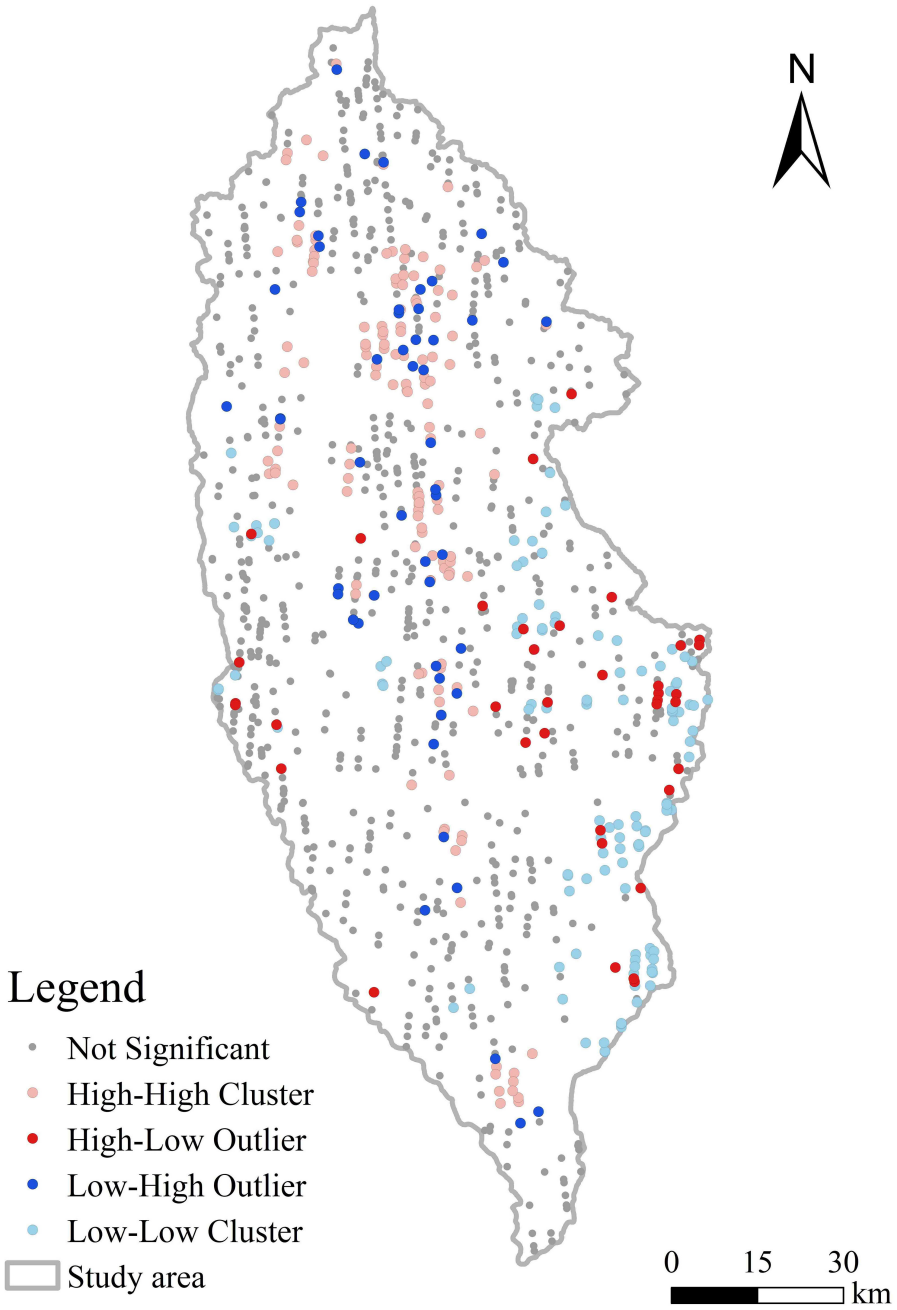
Local spatial autocorrelation of footprint NFCC.

Local spatial autocorrelation analysis can capture local spatial elements’ clustering and difference characteristics. As a common index of local spatial autocorrelation, the local Moran index was used to continue exploring the NFCCs’ spatial relationships of each footprint. As shown in **Figure 10**, NFCC located in the central and northern parts of the study area showed significant HH clustering, while the spatial clustering pattern of the NFCC situated on the west and east sides of the study area showed HL outliers. LH outliers were mainly concentrated in the middle of the study area. Besides, LL clusters were primarily concentrated in the eastern part of the study area.

### 3.4 Spatial heterogeneity of NFCC

**Figure 11:**
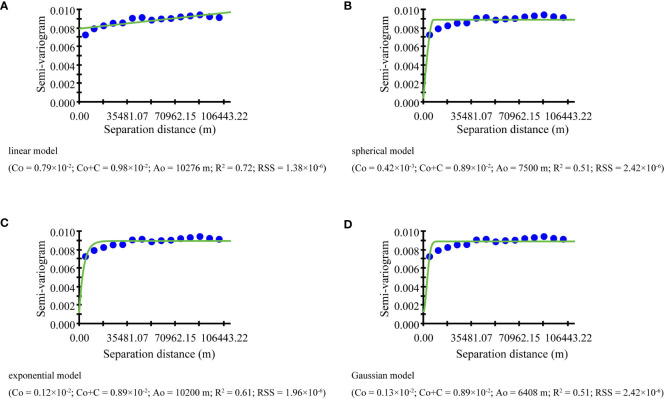
Different mathematical function fits: **(A)** linear model, **(B)** spherical model, **(C)** exponential model, and **(D)** Gaussian model.


The fitting of the semi-variogram function in this study was implemented in GS^+^ 9.0 software. The semi-variogram function revealed the regional differences in the spatial structure of NFCC, and the fitting results were shown in **Figure 11**. According to the principle that the Residual Sum of Squares (RSS) is minimum and the coefficient of determination (R^2^) is maximum, the exponential model (R^2^ = 0.61, RSS = 1.96×10^6^) is best fit to describe the relationship between values and distances. The abutment value (C_0_ + C) of the exponential model is 0.89×10^-2^, the partial sill value (C) is 0.77×10^-2^, the variable range (A_0_) is 10200 m, the nugget value (C_0_) is 0.12×10^-2^, and. The NSR of NFCC is 13.40%, indicating that the variables have strong spatial autocorrelation within the range. The expression of the exponential model [Equation (11)] is as follows:

(11) $$\gamma (h)=\{\begin{array}{ccc} \;0 & , & h=0\\ 0.12\times 10^{-2}+0.77\times 10^{-2\;}\left(1-e^{-\frac{h}{10200}}\right) & , & h>0 \end{array}$$

**Figure 12:**
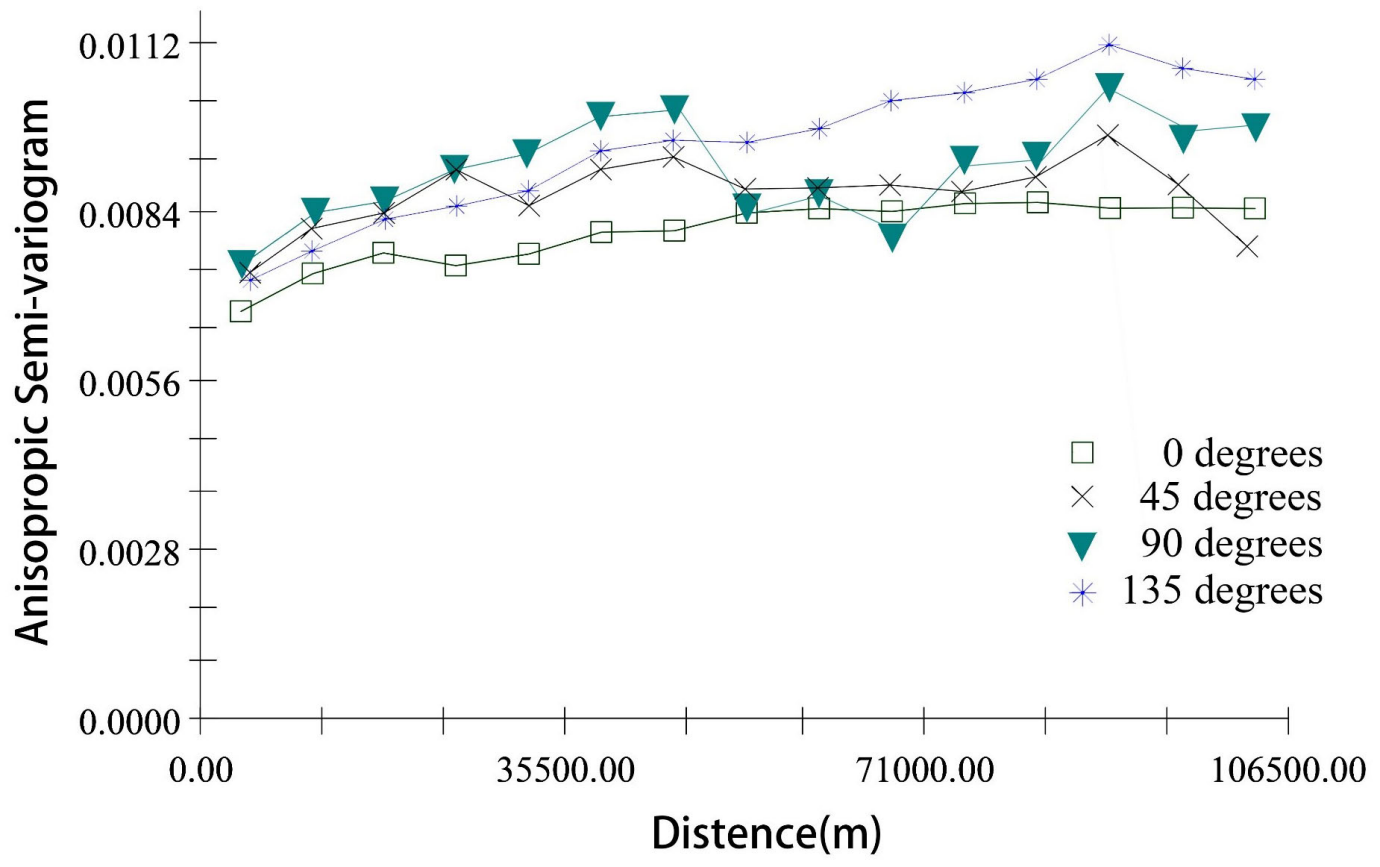
Anisotropic semi-variogram of NFCC in four directions: east-west (0°), south-north (90°), northeast-southwest (45°), and northwest-southeast (135°) in Shangri-La.

The results of the anisotropic semi-variogram function showed that the NFCC changes in all directions on the scale of 106 km (**Figure 12**). The anisotropy of NFCC in the northwest-southeast direction (θ = 135°) was the most obvious, followed by the north-south direction (90°). However, the anisotropy of NFCC in the east-west (0°) direction was relatively low.

### 3.5 Relationship between NFCC and topographic factors

To realize the influence degree of topography on NFCC, Pearson correlation analysis was conducted between the NFCC and the topographic factors (**Table 6**). **Table 6** showed that the NFCC in the study area is significantly correlated with elevation, slope, and aspect at 0.01 level. The order according to the correlation coefficient’s absolute value absolute value of the correlation coefficient was as follows: elevation > slope > aspect.

**Table 6:** Correlation analysis between NFCC and topographic factors.

Variable	NFCC	Elevation	Slope	Aspect
NFCC	1			
Elevation	0.27^**^	1		
Slope	0.10^**^	-0.18^**^	1	
Aspect	0.08^**^	0.02	0.03	1

Signif. Codes: ‘**’0.01; ‘*’0.05. The same below.

### 3.6 Spatial continuous mapping of NFCC

The interpolation results showed that the spatial distribution map of NFCC obtained by the OK method (**Figure 5A**) was roughly similar to the SGCS interpolation map (**Figure 5B**). High values were concentrated mainly in the study area’s northern, central, and southern regions. As can be seen from **Figure 5A**, the spatial distribution of NFCC obtained by the OK method is relatively continuous and has obvious smoothing effect. In **Figure 5B**, the overall distribution using the SGCS method is relatively discrete and less affected by smoothing effect. In addition, the spatial predicted values obtained by SGCS were in good agreement with NFCC footprint values (**Figure 5D**, R^2^ = 0.59). In contrast, the spatial predicted values obtained by OK were less consistent with the NFCC footprint values (**Figure 5C**, R^2^ = 0.43).

## 4 Discussion

### 4.1 Sample size problem for the estimation of NFCC

The results of this study confirm that the combination of ATLAS data, plot survey, ML algorithm, and geostatistical method can provide a valuable framework for county-scale NFCC spatial effects analysis. Before the spatial effects analysis, the NFCC of 1106 footprints was estimate by the RF model. As the input of the model, the measured sample plots play an important role in the modeling. In general, the more samples used, the more reliable the model. However, Shangri-la has many high-altitude mountains and complex terrain, which makes it not easy to collect samples based on LiDAR footprints. In addition, the existing remote sensing estimation of forest parameters is based on the traditional empirical sample size, that is, below 30 is a small sample size, and above 50 is a large sample size (Shu et al., 2022). To minimize the labor and time required, exploring the optimal sample data is needed. Shu et al (Shu et al., 2022). solved the optimal sample size by integrating the statistical variance function and value coefficient, which was reconstructed using the model accuracy evaluation index RMSE and the model sample cost. Therefore, the optimization of the sample size can be further performed in the future to minimize costs.

### 4.2 Uncertainty analysis of the model

Although the number of sample plots is small, the prediction accuracy of the estimation model based on the measured samples was great. However, the phenomenon of high underestimation in all models is relatively easy to find. From the scatter plot (**Figures 7A–D**), when the NFCC is above 0.7, the predicted value below the 1:1 line can be visually seen, which means that the model is still underestimating at higher NFCC. Previous studies (Xing et al., 2010; Xi et al., 2022) have shown that incorporating distinct forest types into the modeling process can enhance performance and decrease the model’s reliance on training samples. However, because of the absence of sample plot data, it was impossible to distinguish between forest types or NFCC levels for modeling. In order to reduce uncertainties in the modeling process, it is recommended that sufficient sample plot data be collected in the future. Furthermore, the importance of physical geography, bioclimate, and biology in estimating forest parameters has been demonstrated (Su et al., 2016; Fayad et al., 2021). Therefore, in future studies, it is suggested that remote sensing data should be combined with a forest physiological process model to enhance the generalization and accuracy of the predictive model.

### 4.3 Spatial distribution characteristics of NFCC

The spatial effect analysis of NFCC obtained by combining machine learning algorithms, relatively new remote sensing data sources, and measured samples is one of the innovations of this study. Many spatial heterogeneity studies (Yao et al., 2015; Li et al., 2017; Liu et al., 2018) can only be carried out on a small scale due to labor costs, resulting in little difference in environmental factors. But the spatial heterogeneity of NFCC is often the result of the interaction of topography, climate, soil, stand, external disturbance, and other random factors. The distribution of light, temperature, water, and other climatic factors is determined by topographic differences. Analyzing the influences of topographic factors on the spatial heterogeneity of forest parameters can provide a better understanding of the mechanism of climate-forest interaction, which is often overlooked in current studies.

The results of this study showed that the canopy cover of Shangri-la natural forest is moderately variable, indicating that it is susceptible to structural and random factors. Spatial variation is mainly divided into structural factor variation and random factor variation (Zou et al., 2021). The NSR of NFCC is 0.13 in section 3.4, showing strong spatial autocorrelation, indicating that the influence of natural factors was dominant. Since 1999, the China National Forestry and Grassland Administration has implemented many large-scale forest conservation projects, such as the Natural Forest Protection Project and Grain for Green Project. Shangri-La is the key area for the implementation of these projects. The natural forests are less disturbed by human factors.

With the support of large-scale spaceborne LiDAR data, this study found that the elevation itself has the most significant influence on the spatial distribution of NFCC, followed by the slope and aspect. However, this study lacks the relationship between topography and climate factors and their joint influence on the spatial distribution of NFCC, which needs further analysis in the future.

### 4.4 Spatial prediction problem based on footprint

As shown in **Figure 8**, with the assistance of ATLAS, the ability to estimate large-scale NFCC is obtained, which is limited to the predetermined ground track footprint range. In view of the feature of ATLAS discontinuous sampling, the discontinuous spatial attributes (NFCC) were used to analyze spatial effects, and extend to the extent of natural forest land throughout the study area by the spatial statistical method. Because spatial interpolation uses known spatial attributes for prediction, the limitations (e.g., climate effect, saturation effect) associated with the using optical images are significantly eliminated (Liu et al., 2022; Yu et al., 2023).

However, the spatial interpolation based on LiDAR spot footprint still faces many problems, such as banding effect, and smoothing effect. Compared with OK, the SGCS method overcomes the shortcomings of Kriging’s smoothing effect (Luo et al., 2023). However, since the spot footprints are distributed along the track, the spatial interpolation results located around the track may have a strong banding effect. Increasing the randomness of spot footprint distribution often has a great effect on avoiding banding effect. In this study, spatial interpolation based on the 1106 footprints obtained through systematic sampling from 11,060 footprints located within natural forests did not show a significant banding effect. Therefore, the sampling of the footprint can be used as an alternative scheme to increase the randomness of the footprint. In addition, Liu et al (Liu et al., 2022). integrated ICESat-2 and GEDI data to carry out spatial interpolation of forest canopy height, and the interpolation results did not show obvious banding effect. Therefore, adding other spaceborne LiDAR data sources can also avoid banding effects.

### 4.5 Prospect of spatial effects analysis of canopy cover based on spaceborne LiDAR data

In this study, the research object only focuses on the NFCC in Shangri-La. Still, the proposed method can be extended to other areas or forest parameters after the same treatment. The Earth will be observed further by ICESat-2/ATLAS, yielding additional high-precision orbital observation data. In order to obtain more and denser space observation footprints, another spaceborne LiDAR named GEDI (Global Ecosystem Dynamics Investigation) can be introduced in future research.

## 5 Conclusions

(1) Among the NFCC prediction models based on 4 ML algorithms (KNN, SVM, GBRT, and RF), GBRT and k-NN are the models with the best and worst prediction performance. The ascending order of predictive performance of the four models is as follows: k-NN (R^2^ = 0.43, RMSE = 0.15), SVM (R^2^ = 0.45, RMSE = 0.14), GBRT (R^2^ = 0.60, RMSE = 0.12), RF (R^2^ = 0.75, RMSE = 0.09).
(2) The results of spatial autocorrelation analysis showed that the NFCC in the study area had a positive spatial correlation, which belonged to the spatial agglomeration distribution. The results of semi-variogram analysis showed that the exponential model is the most suitable to describe the spatial variation characteristics of NFCC (R^2^ = 0.61, RSS = 1.96×10^-6^). The spatial distribution of NFCC in the range of 0~10200 m had a strong spatial correlation.
(3) The spatial heterogeneity of NFCC in the study area is affected by topographic factors. In terms of influence degree, the elevation was the largest, slope was the second, and aspect was the least. In managing natural forests, the function of topographic factors should be considered to manage natural forest scientifically and effectively.
(4) In the spatial distribution maps drawn by OK and SGCS, the spatial distribution obtained by SGCS was in great agreement with the footprint NFCC (R^2^ = 0.59), and was less affected by the smoothing effect.

## Data availability statement

The raw data supporting the conclusions of this article will be made available by the authors, without undue reservation.

## Author contributions

JY: Conceptualization, Formal analysis, Investigation, Methodology, Validation, Visualization, Writing – original draft, Writing – review & editing. LiX: Investigation, Methodology, Visualization, Writing – review & editing. QS: Funding acquisition, Methodology, Project administration, Resources, Supervision, Writing – review & editing. SL: Data curation, Investigation, Visualization, Writing – review & editing. LeX: Data curation, Investigation, Visualization, Writing – review & editing.
